# Reply to Weiss, H.-R. Comment on “Costa et al. The Effectiveness of Different Concepts of Bracing in Adolescent Idiopathic Scoliosis (AIS): A Systematic Review and Meta-Analysis. *J. Clin. Med.* 2021, *10*, 2145”

**DOI:** 10.3390/jcm11040918

**Published:** 2022-02-10

**Authors:** Lorenzo Costa, Tom P. C. Schlösser, Moyo C. Kruyt, René M. Castelein

**Affiliations:** Department of Orthopaedic Surgery, University Medical Center Utrecht, 3508 GA Utrecht, The Netherlands; l.costa-2@umcutrecht.nl (L.C.); t.p.c.schlosser@umcutrecht.nl (T.P.C.S.); m.c.kruyt@umcutrecht.nl (M.C.K.)

We would like to thank you for the opportunity to reply to the comments in regard of the letter by Dr. Weiss [1]. We acknowledge the author’s valuable contribution in this field as reflected by the presence of some of his papers in both our systematic review and meta-analysis.

Most of the criticism seems to stem from a disagreement with our conclusion that the night-time brace concept may be comparable to full-time bracing. Several methodological aspects of our search and selection are mentioned as potential bias in favor of night-time braces. We strongly object to this suggestion. This study was done strictly according to the PRISMA statement with a predetermined objective and quality assessment. A threshold for all papers that were included in the meta-analysis was applied.

We do not agree with the argument that individual case analysis is the correct design to compare brace models. The strength of a systematic review and meta-analysis on this topic is that it can answer a question that cannot be posed by individual studies and can improve the precision of the estimation of effect.

During title and abstract screening, both the title and conclusion suggested that this study, “*Is it possible to improve treatment safety in the corset care of scoliosis patients by applying standardized CAD algorithms?*” should not be included [2]. Nevertheless, reading the full paper, it appears that the aim of the manuscript was to study the *effectiveness* (and not the *safety*) of the Gensingen brace, so in that sense we regret that this paper was not selected. Unfortunately, this kind of error is inherent to the methodology of title and abstract screening, which was performed by two independent researchers [3]. Including a paper post hoc would introduce a potential bias and, therefore, should not be done. Besides the methodological inaccuracy, including the paper would lead to an overlap with the population of another study by the same author, from 2017, that we included in both the systematic review and meta-analysis [4].

Our study was compared with a systematic review by Ruffilli et al. which had a similar aim and Rowe et al. that yielded different results [5,6]. However, Ruffilli et al. only selected comparative studies which resulted in a different and much smaller patient sample. The paper by Rowe et al. (1997), was performed more than 20 years ago in a period where much less studies were present in the literature and the methodology had serious limitations including no strict guidelines for inclusion criteria [6].

One of the aims of this work was to study the effect of skeletal maturity on the effectiveness of brace treatment. Therefore, papers with a Risser grade higher than 2 were also included. Indeed, for this analysis, we only included papers that documented the Risser grade. In the other analyses of effectiveness of braces, all maturation parameters such as menarche (but not only) were included. Furthermore, menarche is considered a valuable tool that is often used as mentioned by Richards et al. in 2005 [7]. Moreover, it is important to notice that also studies on full-time braces with Risser 0–3 or 0–4 were included in the systematic review and meta-analysis [8,9].

We are aware of the letter by Potts et al. in 2020 and their concern regarding the Simony et al. paper [10,11]. They were concerned that the Risser stage was not assessed. Nevertheless, Tanner scale, time of menarche in girls and continued height gain in boys were used [12]. We took both letters into consideration [11,12].

We disagree with the claim that because timing is a key point for brace treatment, it is not possible that the Simony paper has such a positive result [10]. Other studies on night time bracing (which did include Risser sign as a parameter of maturity) showed similar good results [13,14,15]. Therefore, Simony paper is not the first nor the only one. We agree that this result is in the higher range [10]. However, with sufficient quality, the reported outcome cannot be a reason to discard a paper.

The author expresses concern about the variability of the Providence results. We agree that high variability generally weakens the reliability. However, this high variability is not a prerogative of night-time brace concept, but more a general problem of brace studies as was reported in the discussion sections by Ruffilli et al., our article and mentioned by the author himself (specifically focusing on the Cheneau brace) [2,5].

According to the *JCM* Guidelines for authors, “*authors must disclose all relationships or interests that could inappropriately influence or bias their work*”. The analysis was performed according to the above-described and pre-established criteria. One of the authors has received a research grant for studies into scoliosis etiology, but this is unrelated to the current project. Furthermore, the senior author recently founded the Dutch Scoliosis Center. In retrospect, it would have been better to mention this new disclosure, although it had no influence on this particular study. As highlighted in the funding section, this study was supported by funding from the EU’s H2020 research and innovation program under Marie S. Curie cofound RESCUE grant agreement No 801540.

To conclude, we would like to thank the author for highlighting the findings of his previous work and showing his concerns in matter of our systematic review. Nevertheless, we believe that the study was conducted according to widely accepted pre-determined criteria and shows a valid result. This conclusion should not be interpreted as an absolute statement, but is meant to bring awareness in the limitations of the current literature and possibilities of these devices. As we wrote in our discussion, further studies with higher criteria and standards should be done in order to better understand the best treatment and management for AIS patients.

## References

[1] Weiss H.-R. (2022). Comment on Costa et al. The Effectiveness of Different Concepts of Bracing in Adolescent Idiopathic Scoliosis (AIS): A Systematic Review and Meta-Analysis. *J. Clin. Med.* 2021, *10*, 2145. J. Clin. Med..

[2] Weiss H.-R., Lay M., Seibel S., Kleban A. (2021). Is it possible to improve treatment safety in the brace treatment of scoliosis patients by using standardized CAD algorithms?. Orthopade.

[3] Wang Z., Nayfeh T., Tetzlaff J., O’Blenis P., Murad M.H. (2020). Error rates of human reviewers during abstract screening in systematic reviews. PLoS ONE.

[4] Weiss H.-R., Tournavitis N., Seibel S., Kleban A. (2017). A Prospective Cohort Study of AIS Patients with 40° and More Treated with a Gensingen Brace (GBW): Preliminary Results. Open Orthop. J..

[5] Ruffilli A., Fiore M., Barile F., Pasini S., Faldini C. (2021). Evaluation of night-time bracing efficacy in the treatment of adolescent idiopathic scoliosis: A systematic review. Spine Deform..

[6] Rowe D.E., Bernstein S.M., Riddick M.F., Adler F., Emans J.B., Gardner-Bonneau D. (1997). A Meta-Analysis of the Efficacy of Non-Operative Treatments for Idiopathic Scoliosis. J. Bone Jt. Surg..

[7] Richards B.S., Bernstein R.M., D’Amato C.R., Thompson G.H. (2005). Standardization of Criteria for Adolescent Idiopathic Scoliosis Brace Studies: SRS Committee on Bracing and Nonoperative Management. Spine.

[8] Xu L., Yang X., Wang Y., Wu Z., Xia C., Qiu Y., Zhu Z. (2019). Brace Treatment in Adolescent Idiopathic Scoliosis Patients with Curve Between 40° and 45°: Effectiveness and Related Factors. World Neurosurg..

[9] Aulisa A.G., Giordano M., Toniolo R.M., Aulisa L. (2020). Long term results after brace treatment with PASB in adolescent idipathic scoliosis. Eur. J. Phys. Rehabil. Med..

[10] Simony A., Beuschau I., Quisth L., Jespersen S.M., Carreon L.Y., Andersen M. (2019). Providence nighttime bracing is effective in treatment for adolescent idiopathic scoliosis even in curves larger than 35°. Eur. Spine J..

[11] Potts M.A. (2020). Letter to the editor concerning ‘Providence nighttime bracing is effective in treatment for adolescent idiopathic scoliosis even in curves larger than 35°’ by Simony A, Beuschau I, Quisth L; et al. (Eur Spine J; [2019]. https://doi.org/10.1007/s00586-019-06077-z). Eur. Spine J..

[12] Simony A. (2020). Answer to the Letter to the Editor of M. A. Potts concerning “Providence nighttime bracing is effective in treatment for adolescent idiopathic scoliosis even in curves larger than 35°” by Simony A, Beuschau I, Quisth L; et al. [Eur Spine J; (2019): https://doi.org/10.1007/s00586-019-06077-z]. Eur. Spine J..

[13] D’Amato C.R., Griggs S., McCoy B. (2001). Nighttime Bracing with the Providence Brace in Adolescent Girls with Idiopathic Scoliosis. Spine.

[14] Price C.T., Scott D.S., Reed F.E., Riddick M.F. (1990). Nighttime Bracing for Adolescent Idiopathic Scoliosis with the Charleston Bending Brace. Spine.

[15] Lee C.S., Hwang C.J., Kim D.-J., Kim J.H., Kim Y.-T., Lee M.Y., Yoon S.J., Lee D.-H. (2012). Effectiveness of the Charleston Night-time Bending Brace in the Treatment of Adolescent Idiopathic Scoliosis. J. Pediatr. Orthop..

